# New separation protocol reveals spray painting as a neglected source of microplastics in soils

**DOI:** 10.1007/s10311-022-01500-2

**Published:** 2022-10-19

**Authors:** Yaqi Xu, Matthias C. Rillig, Walter R. Waldman

**Affiliations:** 1 Institute of Biology, Freie Universität Berlin, Altensteinstrasse 6, 14195 Berlin, Germany; 2 Berlin-Brandenburg Institute of Advanced Biodiversity Research (BBIB), Königin-Luise-Strasse 4-6, 14195 Berlin, Germany; 3 Center of Science and Technology for the Sustainability, Federal University of São Carlos, Sorocaba, Brazil

**Keywords:** Paint microplastic, Spray paint, Infrared, Separation

## Abstract

Microplastics are recently discovered contaminants, yet knowledge on their sources and analysis is limited. For instance, paint microplastics are poorly known because soil separation protocols using flotation solutions cannot separate paint microplastics due to the higher density of paint microplastic versus common microplastics. Here, we designed a new two-step density separation protocol for paint microplastics, allowing paint microplastics to be separated from the soil without digestion. Paint particles were separated from soil samples collected around the graffiti wall at the Mauerpark, Berlin, then quantified according to their shape and color characteristic. The presence of polymers as binders in the paint particles was verified by Fourier transform infrared spectroscopy. Results show concentrations from 1.1 × 10^5^ to 2.9 × 10^5^ microplastics per Kg of dry soil, representing the highest microplastic concentration ever reported in the literature. Particle concentrations decreased and the median size increased with soil depth. Our results provide first evidence that spray painting, a technique with a wide range of applications from industry to art, leaves a legacy of environmental microplastic in soils that has so far gone unnoticed.

**Supplementary Information:**

The online version contains supplementary material available at 10.1007/s10311-022-01500-2.

## Introduction

Microplastics are a highly diverse group of pollutants from a wide variety of sources with a large variety of shapes and sizes. Examples of common shapes are beads, fibers, foam, fragments, and sheets, ranging in size from a few millimeters to a few microns. In terms of composition, common types of microplastics include, among others, polyethylene, polypropylene, polyamides, polystyrene, polyvinyl chloride, with densities ranging from 0.28 to 1.47 g cm^−3^ (Hanvey et al. 2017). As microplastics are such a broad contaminant suite with different sizes, shapes and densities, this poses particular challenges for environmental detection in complex matrices, such as soil (Rochman et al. 2013; Rillig et al. 2019; Bank et al. 2021).

In this study, we focus on paint as a potential source of microplastics in soil. Paints are considered one of the sources of microplastics because they contain polymer binders as key ingredients (Gaylarde et al. 2021; Song et al. 2014; Turner 2021). Moreover, due to the heavy metals and additives they contain, more attention should be paid to paint microplastics.

Previous studies focused on certain sources of paint particles such as paint peeled off from ships, or fragments of architectural coating and thermoplastic road-surface marking paints that entered urban stormwater drainage systems (Gaylarde et al. 2021; Cunningham et al. 2020; Horton et al. 2017). In fact, most research on microplastics in paint has focused on the aquatic environment or sediments. However, the fate of paint particles in the terrestrial environment is largely unknown, and in particular compared to other common microplastics (Gaylarde et al. 2021).

Spray painting is widely used in graffiti, furnishing painting and industrial spraying. Since the mass transfer efficiency of spray painting usually ranges from 50 to 65%, the remaining material is lost to the air in the form of droplets and aerosols, generating a range of particulates (Heitbrink et al. 1996; Poozesh et al. 2018). For our study, we chose a survey approach in an urban context as a likely site with paint-derived microplastic presence in soil. Mauerpark in Berlin, Germany, is a free urban park that has been open to the public for decades, where countless spray paint artworks have been created and then later covered by new ones on the graffiti wall; the former wall having divided East and West Berlin. During the continuous process of spraying, droplets or aerosols from the spray paint bottles and fragments peeled off from the wall likely have accumulated in the soil over decades. The paint particles found here are colorful, so they lend themselves to visual identification and quantification.

However, paint is denser than most of the microplastics usually monitored in the field, so they are typically not captured by conventional microplastic density separation protocols (Gaylarde et al. 2021; Haave et al. 2019; Schell et al. 2021). Therefore, we devised a new separation protocol specifically focusing on the density range of paint microplastics to enable their quantification in soil.

In the present study, through a sampling survey of soil near the graffiti walls of Mauerpark in Berlin, Germany, we documented high potential for accumulation of paint particles in the soil using our new method, and verified the presence of polymers as binders in paint microplastics by FTIR.

## Materials and methods

On September 30, 2020, soil samples were collected around the graffiti wall at the Mauerpark, Berlin, Germany. Locations *L*1–*L*3 were sampled 2 m away from the wall, whereas locations *L*4–*L*6 were sampled 7.1 m away from the wall (avoiding a concrete sidewalk). Geolocalization is available in the supplementary materials (Table S1). We used a soil drill to collect samples up to 30 cm, and all samples were divided into layers of 5-cm. All soil samples were air-dried and then sieved through a 2-mm sieve.

### Microplastics separation

#### Removing organic matter and less dense microplastics

We used a sodium bromide (NaBr) solution (1.4 g mL^−1^) to remove the less dense microplastics and part of the organic matter by density. Sieved soil (10 g) and NaBr solution (30 mL) were added into each Falcon tube. After vortexing and decantation, the supernatant was discarded, taking care to avoid loss of the soil. This step was carried out twice.

#### Separating the paint microparticles by flotation

To separate the paint microparticles from the soil left in step 1, we used 20-mL mixed salt solution of sodium bromide (NaBr) and zinc chloride (ZnCl_2_) with a final density of 1.8 g mL^−1^. After shaking for 30 min and centrifuging at 2500 G for 30 min, the supernatants were filtered using a vacuum filtration system with a filter with pores of 0.45 µm (Merck Millipore, USA). After the filtration, the filter was observed and analyzed by a LEICA M165C stereomicroscope.

### Microplastics identification

The microplastic nature of the paint fragments in the soil was verified by FTIR. Detailed methods are included in the supplementary materials.

### Microplastics quantification

After the density separation, we observed and photographed the samples using a Leica M165C stereomicroscope. Pictures were taken with the same magnification to avoid the omission of particles of small sizes. Ten pictures were taken randomly in each layer, and colored and spherical or multilayered particles were counted as paint particles. All the data and pictures used for the counting step are available in the supplementary materials. Then, we quantified paint particles using the following formula (Eq. 1):

1 $$N_{1} = \frac{{N_{2} * S_{1} }}{{S_{2} * m_{s} }}$$

where *N*_1_ = Number of paint particles g^−1^ (dry soil); *N*_2_ = Number of paint particles in a given microscope field; *S*_1_ = Area of the filter with soil (here always 1017.36 mm^2^); and *S*_2_ = Area of microscope field in a given magnification (here always 0.035 mm^2^); *m*_s_ is the mass of each soil sample (here always 10 g).

## Results and discussion

### Extraction of paint particles from soil

This work aims to monitor microplastics in soil derived from spray painting, so we developed a strategy to use the higher density of these particles to separate them from the soil and from the potential other microplastics that are present as well. The most studied microplastics, like polypropylene, low-density polyethylene, polystyrene, polyvinyl chloride, polybutylene, and polylactic acid, all float using NaBr solutions (density of 1.4 g cm^−3^), whereas microplastics from spray painting, which are denser because of the pigments inside, sink in this density range (Li et al. 2021; Quinn et al. 2017; Turner et al. 2021). For instance, Tafuri et al. (2012) determined the density of paint applied in ship painting processes as 1.69 g cm^−3^. To separate the paint particles after the first separation with NaBr, a denser brine solution is required. ZnCl_2_ solution can provide densities up to 1.8 g cm^−3^ (Haave et al. 2019; Bergmann et al. 2017), but ZnCl_2_ is expensive and hazardous (Bergmann et al. 2017; Cutroneo et al. 2021; Haave et al. 2019; Han et al. 2019). To reduce the usage of ZnCl_2_, a mixed solution of NaBr and ZnCl_2_ was prepared from a saturated NaBr solution to which we added ZnCl_2_ up to the density of 1.8 g cm^−3^. This protocol removed organic matter and the less dense microplastics in the first step using the NaBr solution (1.4 g cm^−3^) and then separated the denser paint particles using the NaBr and ZnCl_2_ mixed solution (1.8 g cm^−3^).

**Fig. 1:**
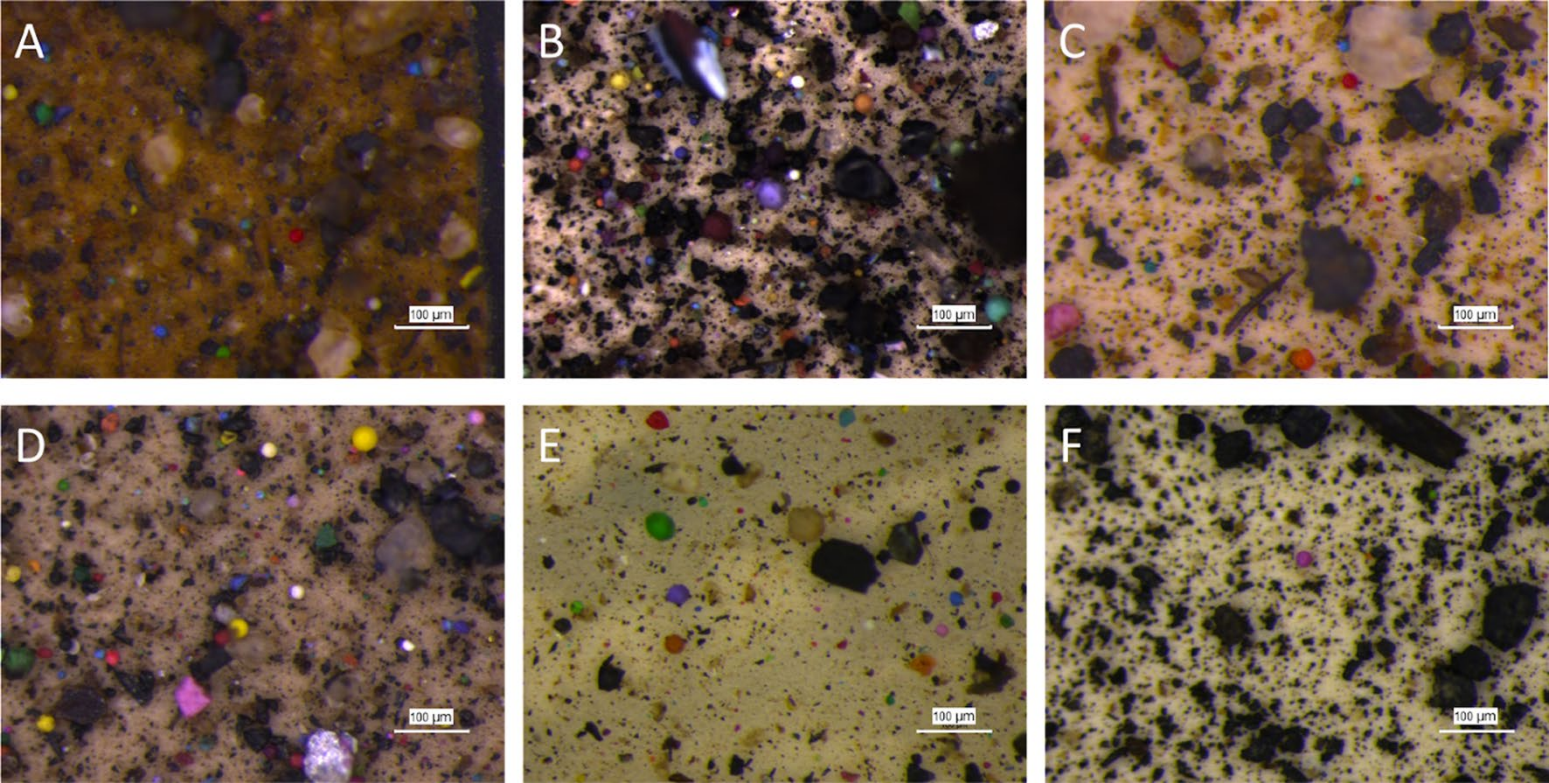
Micrographs of paint microplastics, visible as colorful objects against the soil material background, separated from the 0–5-cm layer soil samples from the six sampling locations (**A**: Location 1; **B**: Location 2; **C**: Location 3; **D**: Location 4; **E**: Location 5; **F**: Location 6) near the graffiti wall in Mauerpark (Berlin, Germany). The size bar represents 100 µm. Micrographs of paint microplastics in deeper layers of soil are available in the supplementary materials (Fig. S1)

The pictures of the fraction produced after the separation protocol for all the 0–5-cm soil layers are shown in Fig. 1. (Figure S1 in the supplementary materials shows pictures representative of the microplastics for all the layers in the six collection locations.) In all samples, we can see characteristic, mostly spherical and colored, paint particles formed from the spray aerosols that were deposited onto the soil close to the wall. The darker mass in the background is organic matter denser than the first step of separation but less dense than the second separation solution.

**Fig. 2:**
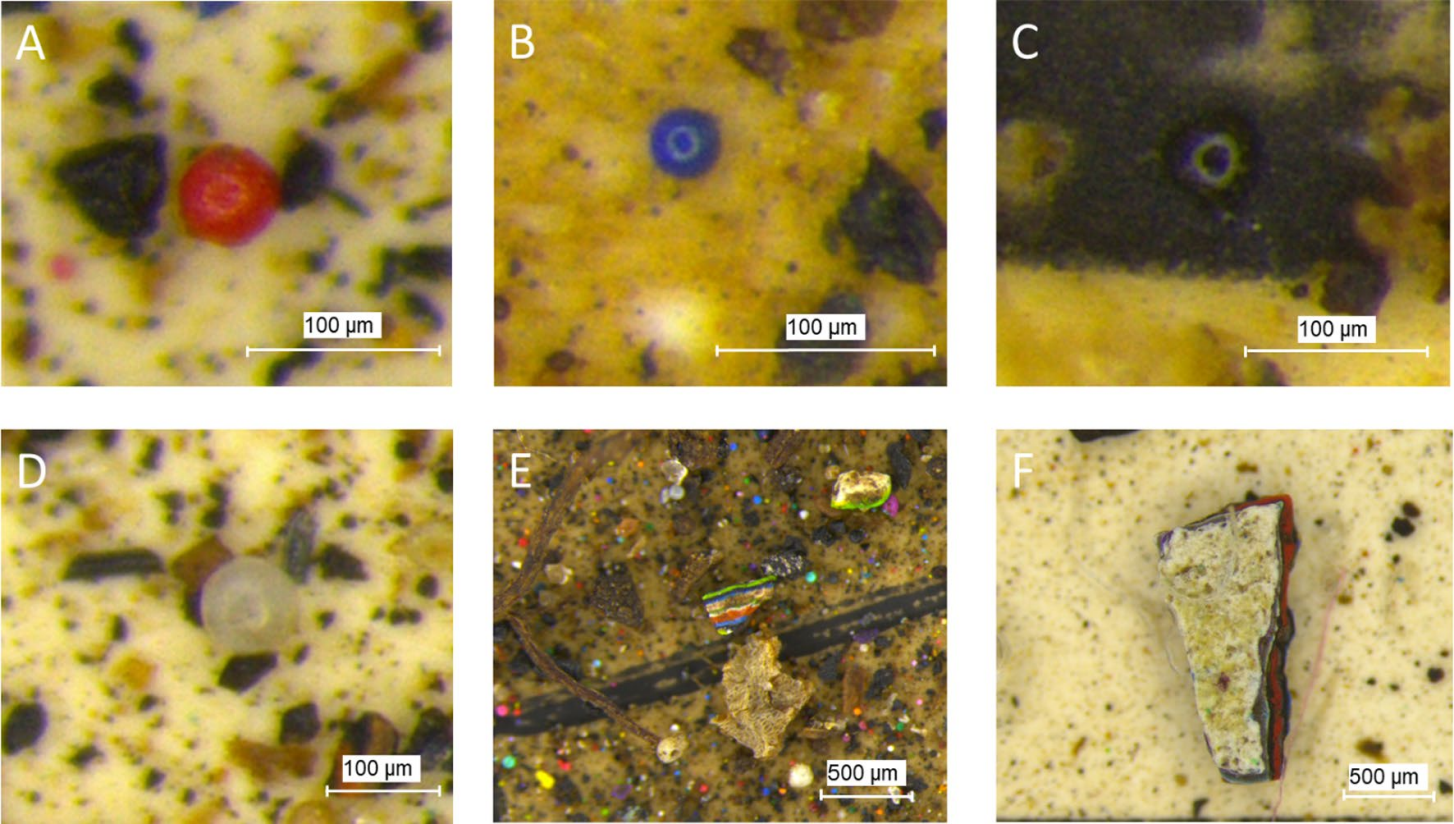
Micrographs of spherical paints separated from the soil samples collected near the graffiti wall in Mauerpark (Berlin, Germany) with a distinctive reflection phenomenon (**A**–**D**) and examples of multiple layered paints found in the collected soil samples worn out from the graffiti wall (**E**–**F**). The size bar represents 100 µm (**A**–**D**) and 500 µm (**E**–**F**)

The most obvious identifying feature of the paint particles is their intense color. The existence of microplastics with colors not visible given the background color could lead to an underestimate of particle numbers. However, spherical paint particles feature a distinctive reflection phenomenon due to their shape, resulting in a light circle in the middle of the particles (Fig. 2A, B), contributing to identifying dark (Fig. 2C) or lower contrast (Fig. 2D) particles.

In addition to spherical particles produced from droplets and aerosols, some paint fragments have multiple-colored layers (Fig. 2E, F). These fragments were probably peeled or worn from the graffiti walls and tend to have a larger size than the fragments that originated directly from droplets or aerosols. For example, Fig. 2 F shows the fragment with the largest size of 1.56 mm among all paints found in the six sampling locations.

### Additional verification of microplastics

Since most of the plastics used in paints are completely or partially soluble in xylene, an often-used component of paint removers, we carried out extraction of the paint binders from the soil using xylene and analyzed the film formed after the solvent evaporated. The film yielded a predominance of alkyd and styrene-acrylic resins and a minor contribution of polyvinyl acetate. They are all polymers typically used as binders in paints (Gao et al. 2020; Liu et al. 2021). The spectra and all the attribution of the absorption bands are shown in the supplementary materials (Figure S3 and S4).

### Quantification and size distribution of paint particles in the soil

**Fig. 3:**
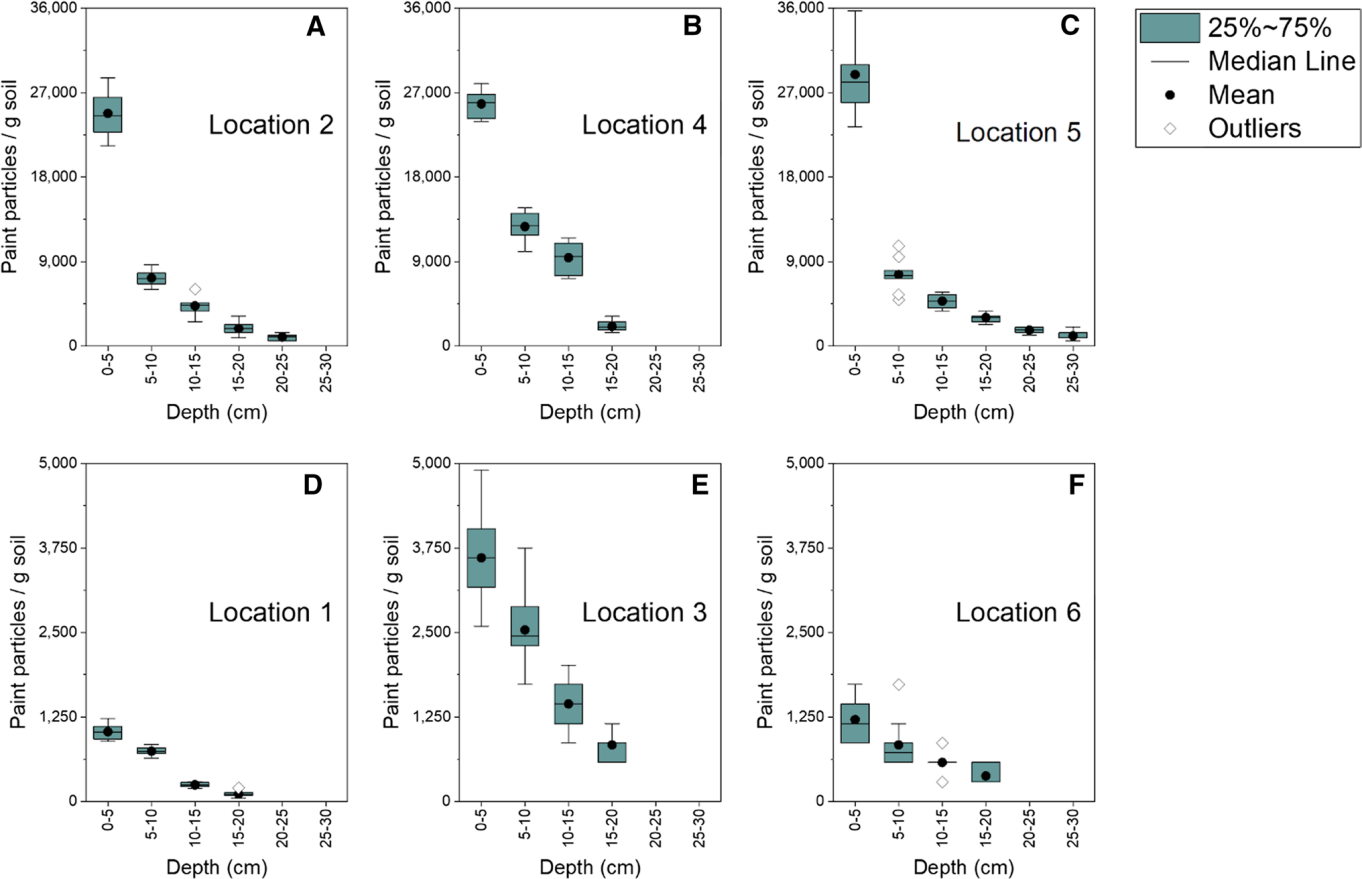
Concentrations of paint microplastics separated from the soil samples as a function of soil depth at different locations next to the graffiti wall in Mauerpark (Berlin, Germany). For each sampling location, at least 4 layers of soil were examined, with a minimum sampling depth of 20 cm and a maximum sampling depth of 30 cm. For all locations, there was clear decrease in the abundance of paint microplastics with soil depth. *Y*-axes have different scales for better comparison. See also Fig. S2 for more detail

We quantified the number of microplastics for each soil layer Eq. 1 (Fig. 3). The highest numbers of paint microplastics among the topmost layers of the different sampling locations vary from almost 30,000 particles g^−1^ to less than 1500 particles g^−1^. This difference is mainly caused by the sampling locations. The high number of particles found in this work can be explained by the dynamics of the Mauerpark graffiti wall. Since spraying paint there is legal and open for all, once the work is done the graffiti can be covered by another one. This characteristic guarantees a sustained delivery of droplets and aerosols to the area close to the wall. Added to the low dispersion rate of the soil, we observe a microplastics accumulation.

The highest concentrations we found were in the topsoil layers (0–5 cm) of three locations: 2.48 × 10^7^ particles kg^−1^ dry soil (Location 2), 2.58 × 10^7^ particles kg^−1^ dry soil (Location 4), and 2.89 × 10^7^ particles kg^−1^ dry soil (Location 5). For the sake of comparison, we investigated recent review papers covering the occurrence of microplastics in soil and selected those which reported the highest concentration. Koutnik et al. (2021) found the highest concentration of 9.5 × 10^5^ microplastics kg^−1^ in the sediment of a stormwater pond; Yang et al. (2021) and Zhou et al. (2020) found the highest concentration of 6.9 × 10^5^ microplastics kg^−1^ in Woodlands; and Gao et al. (2020) and Liu (2021) described the highest concentration of 1.86 × 10^5^ microplastics kg^−1^ in sewage. The concentrations in the top layer of the collected soil close to the graffiti wall in Mauerpark are the highest concentrations of microplastics in soil reported so far.

**Fig. 4:**
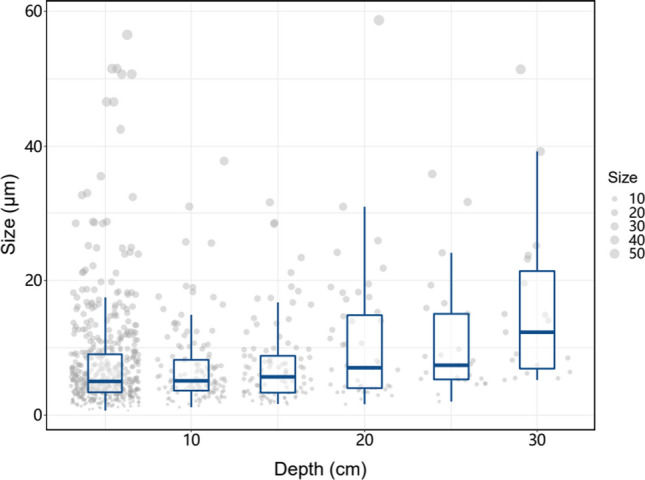
Size distribution of the paint microplastics as a function of soil depth for location 5, showing a shift toward larger microplastic particle sizes with depth. The paint microplastics were separated from the soil sample collected in location 5 near Mauerpark (Berlin, Germany). Boxplots show median, first and third quartiles, with whiskers showing range. Individual data points show sizes of paint microplastic particles (see legend on the right, in µm)

Regarding the size distribution, we chose the location with the highest concentration (Location 5) and assessed the size distribution as a function of the depth. As shown in Fig. 4, median and box plots shifted to larger particle sizes as a function of the depth. This result indicates the likely interaction of the smaller particles with the soil rather than moving toward deeper layers.

Furthermore, such high concentrations of microplastics in the soil will inevitably affect soil properties and activities of soil organisms. Therefore, relevant regulations should be established and applied to outdoor spray painting, including the creation of graffiti arts, to facilitate the recycling of paints and to reduce the input of microplastic paints into the environment.

However, we very likely still underestimated the real number of paint microplastic in soil, as no separation protocol is without deficiencies; here, the following factors may have contributed to a decreased detection efficiency: loss of paints during the separation of organic matter, omission of particles with similar color to the background by visual identification, and detection limits of small size < 4 μm particles due to the limited resolution of the microscope.

## Conclusion

In the present study, we performed a sampling survey of soil near graffiti walls in Berlin, Germany, and analyzed these soils for spray paint microplastic particles, using a protocol we specifically developed for this purpose. Given the large amount of paint microplastic particles we find in this case study, we strongly suggest that the ecological effects of paint microplastics in soil should be a focus of future studies. We also encourage other researchers to follow our protocol and add to the database of paint microplastics in soil; we strongly suggest including this microplastic type in ongoing surveys of microplastics, especially in urban areas. Our findings also suggest that spray painting and similar industrial processes should be regulated in areas where this is not yet the case; in addition, we advocate for monitoring spray painting of larger structures that are impossible to coat in closed spaces in order to reduce the environmental contamination risk.

## Supplementary Information

Below is the link to the electronic supplementary material.

Supplementary file1 (DOCX 2186 KB)

## Data Availability

The datasets that support the findings of this study can be found in online repositories (https://doi.org/10.6084/m9.figshare.20393856).
