# Supplementary material for: New separation protocol reveals spray painting as a neglected source of microplastics in soils
Source: Environ Chem Lett. 2022 Oct 19;20(6):3363–9. doi: 10.1007/s10311-022-01500-2 (PMC9712295; doi:10.1007/s10311-022-01500-2)
Supplement: Supplementary file 1 — Supplementary file1 (DOCX 2186 KB) [file 10311_2022_1500_MOESM1_ESM.docx]

New separation protocol reveals spray painting as a neglected source of microplastics in soils

Yaqi Xu^1,2^, Matthias C. Rillig^1,2^*, Walter R. Waldman^3^*

Affiliations

^1^ Freie Universität Berlin, Institute of Biology, Altensteinstrasse 6, 14195 Berlin, Germany

^2^ Berlin-Brandenburg Institute of Advanced Biodiversity Research (BBIB), Königin-Luise-Strasse 4-6, 14195 Berlin, Germany

^3^ Center of Science and Technology for the Sustainability, Federal University of São Carlos, Sorocaba, Brazil

*Corresponding authors:

Matthias C. Rillig: [rillig@zedat.fu-berlin.de](mailto:rillig@zedat.fu-berlin.de)

Walter R. Waldman: [walter.waldman@gmail.com](mailto:walter.waldman@gmail.com)

1.1 Method of microplastics identification

To verify the microplastic nature of the paint fragments in the soil, we selected the soil sample with the highest concentration to extract the polymers used as binders with xylene, selected due to its efficient performance in cleaning and removing paints. Pieces of graffiti paint peeling off the Mauerpark graffiti wall (2 g), original soil sample from the 0-5 cm layer (10 g), and 0-5 cm soil sample after separation protocol (10 g) were mixed with 5 mL of xylene in glass beakers and sat at room temperature overnight. After filtration through a 0.25 µm vacuum filtration system, the filtered solution was placed in silicone containers in a fume hood to evaporate the xylene. Finally, we analyzed the residual film in the silicone containers using an infrared spectrophotometer Jasco FT/IR 4100 (Jasco International Co. Ltd., Japan), 64 scans, resolution of 4 cm^-1^, from 4,000 to 650 cm^-1^.

1.2 Tables and figures

Table S1 Geolocalization of the pictures taken in each sampling locations near Mauerpark (Berlin, Germany) using metadata.

| Location | Geolocalization - image  (Degrees Lat Long)* |  | Location | Geolocalization - image  (Degrees Lat Long)* |
| --- | --- | --- | --- | --- |
| 1 | (52.5440278°, 013.4008222°) |  | 4 | (52.5435944°, 013.4036556°) |
| 2 | (52.5437250°, 013.4037250°) |  | 5 | (52.5426056°, 013.4039139°) |
| 3 | (52.5443528°, 013.4015389°) |  | 6 | (52.5428472°, 013.4039694°) |

*Converted using <https://www.earthpoint.us/convert.aspx>

Table S2: Attribution of the infrared bands showed in figure S3 and figure S4.

| Wavenumber  (cm^-1^) | Alkyd resin | Styrene-acrylic resin | Polyvinyl Acetate (PVA) | Attribution |
| --- | --- | --- | --- | --- |
| 3063 |  | x |  | C-H stretching (styrene) |
| 3030 | x | x |  | C-H stretching |
| 2956 | x | x |  |  |
| 2923 | x | x |  |  |
| 2870 |  | x |  | C-H stretching (acrylic) |
| 2849 | x |  |  | C-H stretching |
| 1725 | x | x |  | Benzoate ester (alkyd); carbonyl (acrylic) |
| 1600 |  | x |  | styrene and α-methyl-styrene copolymer |
| 1492 | x | x |  | Aromatic ring breathing |
| 1450 | x | x |  |  |
| 1385 |  | x |  | C-H bending |
| 1240 |  |  | x | C-H wagging mode |
| 1150 |  | x |  | C-O (acrylics) |
| 1120 |  |  | x | C-O stretching |
| 1064 | x | x |  | C-C-O asymmetric stretching |
| 1026 |  |  | x | C-O stretching |
| 966 |  | x |  | C-C (acrylics) |
| 747 | x | x |  | Aromatic C-H out-of-plane bending |
| 700 | x | x |  | Aromatic C-H out-of-plane bending |

Table S3 Quantification data of paint microplastics separated from soil samples in 6 locations near Mauerpark (Berlin, Germany)

| Location | Depth (cm) | Paint microplastics/g dry soil |
| --- | --- | --- |
| 1 | 0-5 | 3257.1 |
|  | 5-10 | 2507.6 |
|  | 10-15 | 951.2 |
|  | 15-20 | 345.9 |
| 2 | 0-5 | 24788.2 |
|  | 5-10 | 7234.7 |
|  | 10-15 | 4265.9 |
|  | 15-20 | 1844.7 |
|  | 20-25 | 951.2 |
| 3 | 0-5 | 3602.9 |
|  | 5-10 | 2536.5 |
|  | 10-15 | 1441.2 |
|  | 15-20 | 835.9 |
| 4 | 0-5 | 25797.0 |
|  | 5-10 | 12711.1 |
|  | 10-15 | 9396.4 |
|  | 15-20 | 2104.1 |
| 5 | 0-5 | 28938.8 |
|  | 5-10 | 7609.4 |
|  | 10-15 | 4784.7 |
|  | 15-20 | 3026.5 |
|  | 20-25 | 1671.8 |
|  | 25-30 | 1066.5 |
| 6 | 0-5 | 1210.6 |
|  | 5-10 | 835.9 |
|  | 10-15 | 576.5 |
|  | 15-20 | 374.7 |

Table S4 Size data of paint microplastics separated from soil samples in location 5

| Depth (cm) | Size average (μm) | Median (μm) |
| --- | --- | --- |
| 0-5 | 7.66 | 5.02 |
| 5-10 | 7.01 | 5.09 |
| 10-15 | 7.55 | 5.69 |
| 15-20 | 10.69 | 7.04 |
| 20-25 | 10.69 | 7.41 |
| 25-30 | 15.81 | 12.31 |


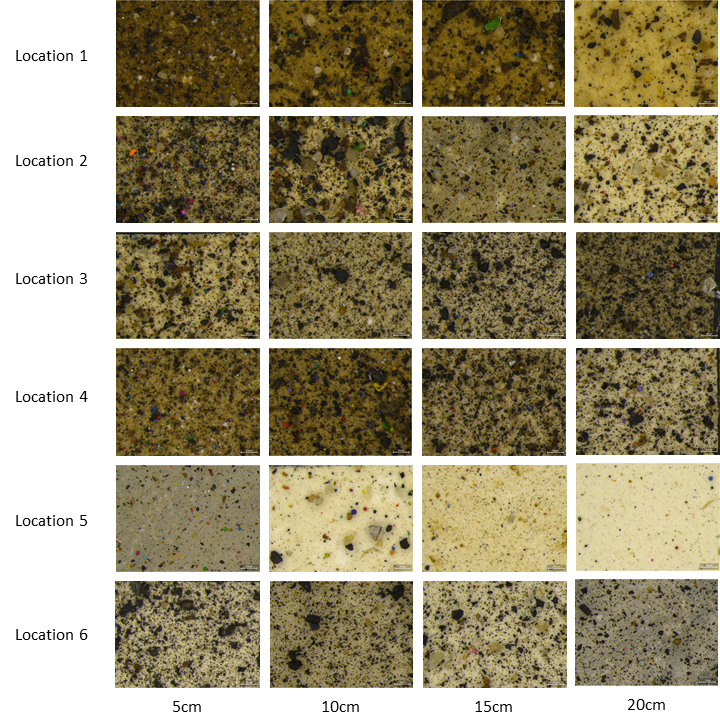


Figure S1. Optical microscopy pictures representatives of the counting for each layer for all the locations under the same magnification. The pictures showed that the number of paint particles decreased as the sampling depth increased. Size bar of each picture is 250 µm.


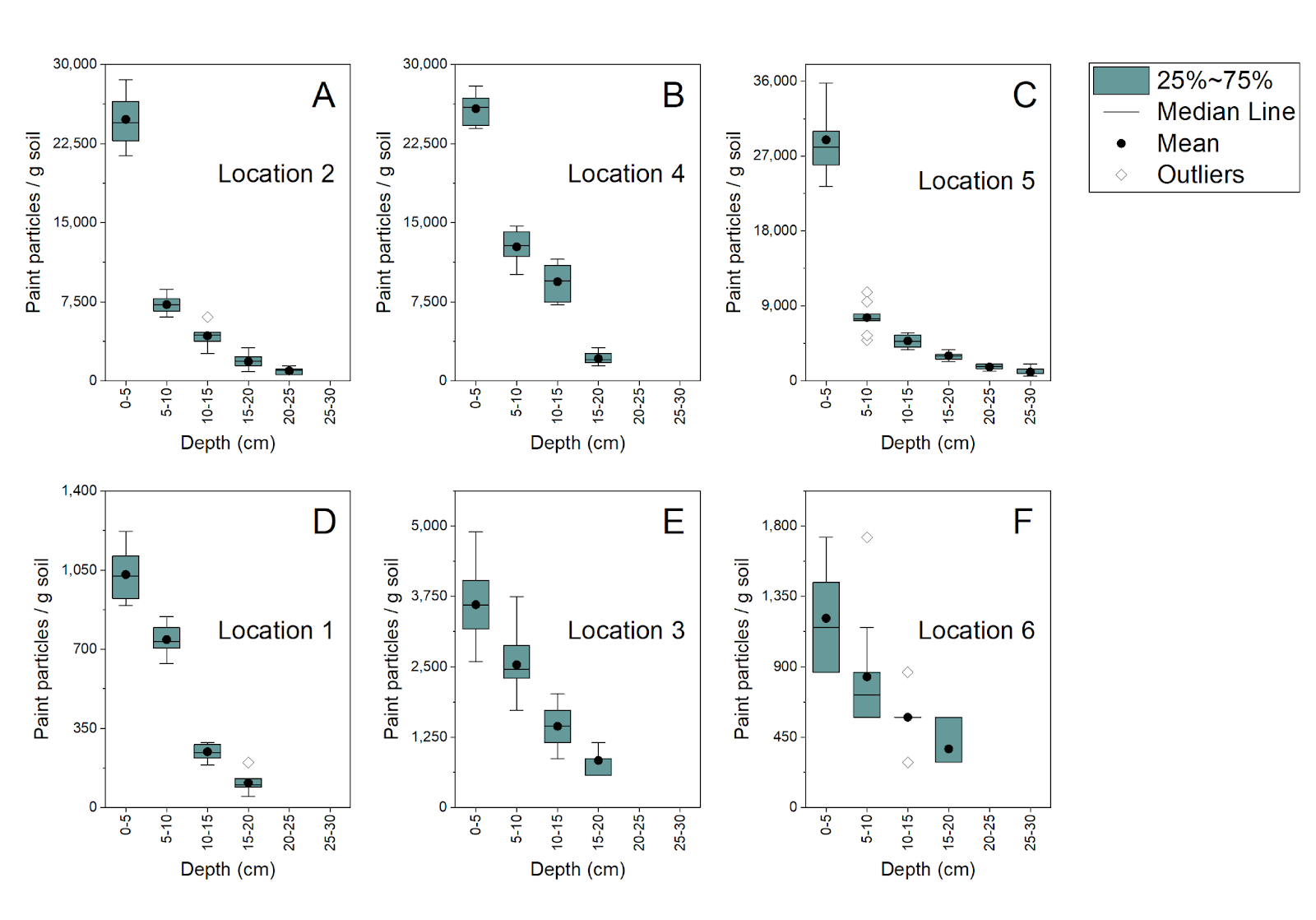


Figure S2. Representation of colored microplastics concentration in different locations of the graffiti wall at the Mauerpark. Quantification was represented with the best scale for each location for better visualization.


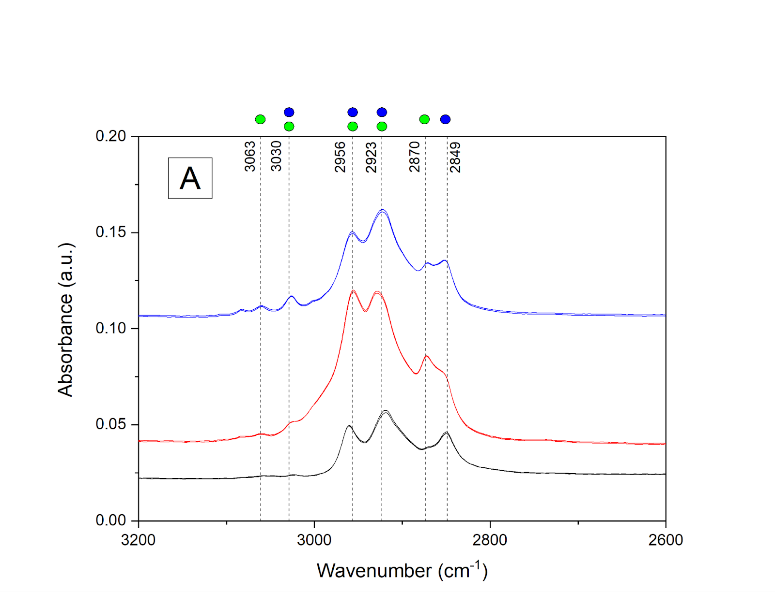

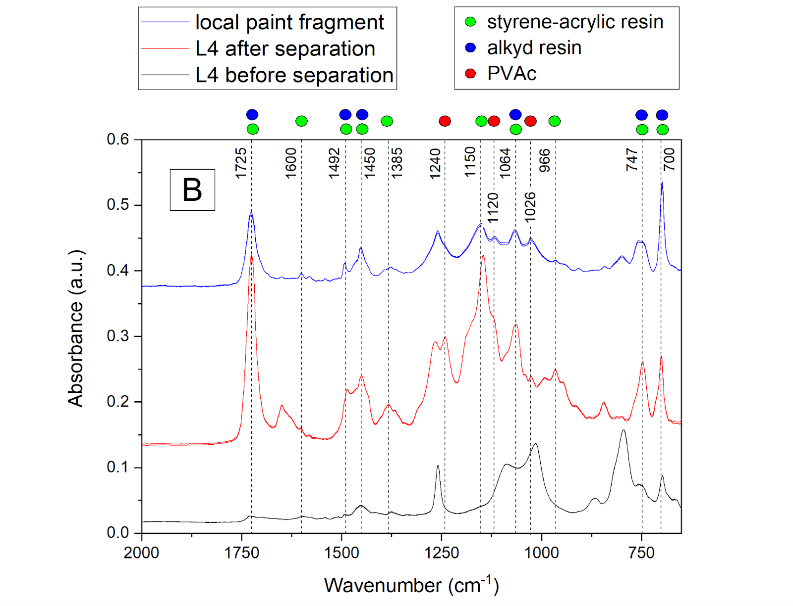


Figure S3. Infrared spectra of the peelings from the Mauerpark wall (blue line); Film from xylene extraction from the 0-5 cm layer of Location 4 before (black line) and after (red line) the separation protocol. A) region between 3200 and 2600 cm^-1^; B) region between 2000 and 700 cm^-1^.


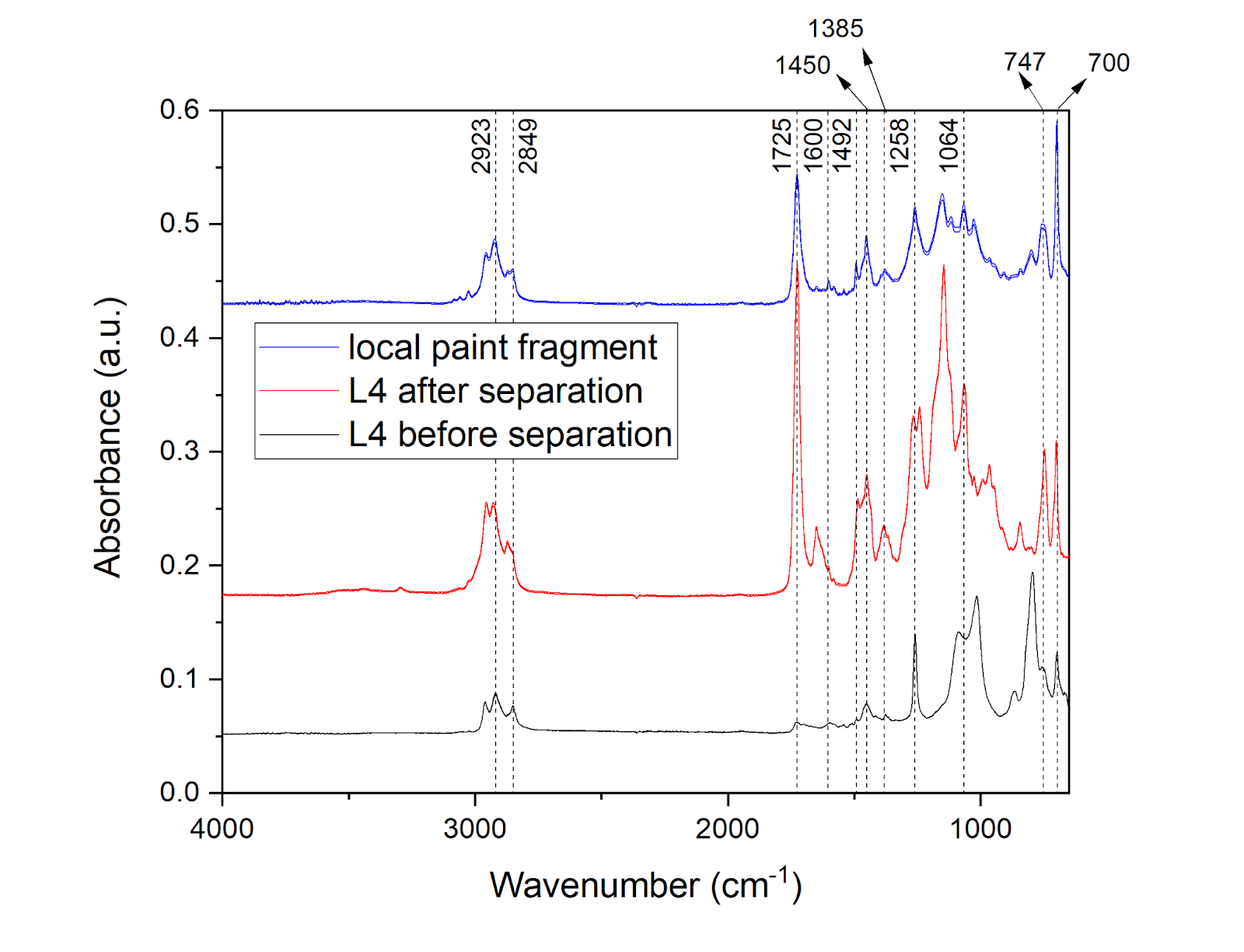


Figure S4. Full extension of ATR-FTIR spectra for the duplicates of the local paint fragment (blue line); solvent extraction from location 4 soil after the separation protocol (red line); solvent extraction from location 4 soil before the separation protocol (black line).
